# Coordinated and Interactive Expression of Genes of Lipid Metabolism and Inflammation in Adipose Tissue and Liver during Metabolic Overload

**DOI:** 10.1371/journal.pone.0075290

**Published:** 2013-09-25

**Authors:** Wen Liang, Giulia Tonini, Petra Mulder, Thomas Kelder, Marjan van Erk, Anita M. van den Hoek, Rob Mariman, Peter Y. Wielinga, Michela Baccini, Teake Kooistra, Annibale Biggeri, Robert Kleemann

**Affiliations:** 1 Department of Metabolic Health Research, TNO (the Netherlands Organization for Applied Scientific Research), Leiden, the Netherlands; 2 Biostatistics Unit, Cancer Prevention and Research Institute, Florence, Italy; 3 Department of Microbiology and Systems Biology, TNO (the Netherlands Organization for Applied Scientific Research), Zeist, the Netherlands; 4 Department of Statistics, Informatics and Applications "G. Parenti", University of Florence, Florence, Italy; Graduate School of Medicine, the University of Tokyo, Japan

## Abstract

**Background:**

Chronic metabolic overload results in lipid accumulation and subsequent inflammation in white adipose tissue (WAT), often accompanied by non-alcoholic fatty liver disease (NAFLD). In response to metabolic overload, the expression of genes involved in lipid metabolism and inflammatory processes is adapted. However, it still remains unknown how these adaptations in gene expression in expanding WAT and liver are orchestrated and whether they are interrelated.

**Methodology/Principal Findings:**

ApoE*3Leiden mice were fed HFD or chow for different periods up to 12 weeks. Gene expression in WAT and liver over time was evaluated by micro-array analysis. WAT hypertrophy and inflammation were analyzed histologically. Bayesian hierarchical cluster analysis of dynamic WAT gene expression identified groups of genes (‘clusters’) with comparable expression patterns over time. HFD evoked an immediate response of five clusters of ‘lipid metabolism’ genes in WAT, which did not further change thereafter. At a later time point (>6 weeks), inflammatory clusters were induced. Promoter analysis of clustered genes resulted in specific key regulators which may orchestrate the metabolic and inflammatory responses in WAT. Some master regulators played a dual role in control of metabolism and inflammation. When WAT inflammation developed (>6 weeks), genes of lipid metabolism and inflammation were also affected in corresponding livers. These hepatic gene expression changes and the underlying transcriptional responses in particular, were remarkably similar to those detected in WAT.

**Conclusion:**

In WAT, metabolic overload induced an immediate, stable response on clusters of lipid metabolism genes and induced inflammatory genes later in time. Both processes may be controlled and interlinked by specific transcriptional regulators. When WAT inflammation began, the hepatic response to HFD resembled that in WAT. In all, WAT and liver respond to metabolic overload by adaptations in expression of gene clusters that control lipid metabolism and inflammatory processes in an orchestrated and interrelated manner.

## Introduction

The obesity epidemic has become the most important nutritional problem worldwide. The increasing prevalence of obesity has been ascribed to excessive and unhealthy eating and reduced physical activity [1] and carries with it increased risks for type 2 diabetes (T2DM) and non-alcoholic fatty liver disease (NAFLD) [2-4]. Notably, the existence of metabolic pathways that allow excess energy to be stored as fat suggests that obesity may realistically be viewed as a biological adaptation in times of energy surplus. However, in case of prolonged excess energy supply white adipose tissue (WAT) capacity may exceeded [5,6] and the liver may serve as an alternative depot for free fatty acids [7,8]. The resulting metabolic overload of the expanding WAT and subsequently liver is accompanied by local metabolic stress and triggers tissue inflammation [9].

Several studies in mice have demonstrated that the expression of genes of lipid metabolism and inflammation is adjusted in several organs in response to chronic high-fat diet (HFD) feeding [10-12]. These studies are often static and focus on a single organ which is analyzed at one particular time point, typically at a later stage in the disease process when histopathological effects of HFD can be detected (e.g. crown-like structures in WAT, inflammatory gut, hepatic steatosis). Accordingly, many gene expression studies and sophisticated microarray analyses link late stage histological outcomes to gene expression changes of the same (late) time point. However, significant effects on gene expression are likely to start early and change over time. Hence, there is limited understanding of the early events in expanding WAT and in what way lipid metabolism is related to the onset of inflammation during obesity development. Some studies investigated the expression changes of *individual* inflammatory genes and pathways over time [13-15], but they did not explore whether groups of genes (

‘gene clusters’) change in concert and thus do not provide insight into the global adaptations and possible common transcriptional regulation of clustered genes.

Bayesian hierarchical cluster analysis [16] allows global analysis of dynamic gene expression data of thousands of genes simultaneously to find patterns in the data that are not predicted by the experimenter’s current knowledge or preconceptions. For instance, complex gene expression time series can be analyzed to identify genes with similar expression patterns that group into clusters because of common transcriptional regulation. Cluster analysis of the expanding WAT requires dynamic high-quality microarray datasets with multiple *early* time points, which are scarce [13]. In the present study we investigated two important processes in WAT expansion using cluster analysis: the global adjustment of genes of ‘lipid metabolism’ and the induction of ‘inflammatory genes’, and their interrelationship. Because transcriptional control mechanisms are instrumental for adjustment of lipid metabolism as well as inflammatory gene expression [17], we examined whether the genes of the identified clusters share common transcriptional regulation, *viz*. via key master regulators.

Metabolic overload of WAT upon HFD feeding is, at a later stage, supposed to be accompanied by multiple metabolic and inflammatory effects in the liver [7]. It is presently unknown whether the effects on genes of metabolism and inflammation are similar to WAT and, if so, whether the same master regulators are involved.

WAT and liver tissues and corresponding dynamic genomics datasets from a 12-week HFD feeding experiment [13] in APOE*3Leiden transgenic mice were used. APOE*3Leiden mice have a humanized lipoprotein metabolism and develop obesity, insulin resistance and NAFLD during HFD feeding [18-20]. Bayesian cluster analysis in conjunction with promoter analysis and biochemical measurements showed that adjustment of lipid metabolism and onset of inflammation in WAT occurs sequentially and is orchestrated by specific master regulators that also control comparable changes in lipid metabolism and inflammation in the liver later in time.

## Materials and Methods

### Mouse study and micro-array data

Tissues and micro-array data from a larger time course study in APOE*3Leiden mice in the context of HFD-induced insulin resistance were used [13]. These micro-array datasets (liver and WAT) are freely available on ArrayExpress at the following URL http://www.ebi.ac.uk/arrayexpress/experiments/E-TABM-1039/. Animal experiments were approved by the Institutional Animal Care and Use Committee of The Netherlands Organization for Applied Scientific Research (TNO), and were in compliance with European Community specifications regarding the use of laboratory animals as reported [13]. Briefly, 12 weeks old mice were fed HFD containing (all w/w) 24% fat from beef tallow (of which 12% saturated fatty acids), 24% casein and 20% dextrose (diet number 4031.05; Hope Farms, Woerden, The Netherlands; metabolizable energy: 19.4 MJ/kg; exact diet composition is provided in Table S1) for 12 weeks [13]. Mice were sacrificed at t=0 and after 1, 6, 9 and 12 weeks of HFD feeding (n=15/group). Epididymal adipose tissue and corresponding livers of a subset of animals (n=8) per time point were used for microarray analysis. Our present data are from this subset of animals. A separate control group (n=6) was fed chow (sniff® R/M-H; metabolizable energy: 12.8 MJ/kg; Sniff Spezialdiäten GmbH, Soest, Germany) for the entire study period and served as a reference for the effect of aging.

### Histological analysis of tissues

Paraffine-embedded sections of adipose tissue and liver were used for (immuno) histological examination [13]. Liver tissue sections were 5 µm thick and stained with hematoxylin phloxine saffron (HPS). Non-alcoholic fatty liver disease was analyzed as described [21] and vacuolization (micro- and macrovaculolization) and hepatocellular hypertrophy were scored. Sections of epididymal adipose tissue were prepared following a similar procedure [22] and stained with HPS for computer-assisted morphological assessment of adipocyte size and analysis of macrophage accumulation in crown-like structures essentially as reported [20]. CCR2 positive cells were detected using antibody (Abcam ab21667, Cambridge, UK).

### Microarray data analysis and Bayesian hierarchical clustering

Quality control analyses and specific protocols for RNA extraction, RNA integrity assessment, microarray data processing were reported previously [13,23]. Briefly, quality control of microarray data was performed using BioConductor packages including simpleaffy and affyplm, through the NuGO pipeline that is available as a Genepattern procedure on http://nbx2.nugo.org [24]. Thirty-eight adipose tissue samples passed the quality control criteria and raw signal intensities (from CEL files) were normalized using the GCRMA algorithm (gc-rma slow). Probesets were remapped and annotated into Entrez gene-ids using the custom MBNI CDF-file, version 9.0.1. The final dataset contained the expression values of 12492 adipose tissue genes represented by unique Entrez gene-ids [25]. Expression data were logtransformed for further analysis of gene expression levels. Microarray gene expression data were confirmed by quantitative real-time PCR for a selection of genes using established protocols and primer/probe sets [13].

For this study, two sets of genes with either lipid metabolism ontology (n=235) or inflammation ontology (n=216) were defined. These genes were differentially expressed at one or more time points (q<0.05ANOVA) and are listed in Table S2. The time course expression data of these genes was subjected to Bayesian hierarchical clustering to structure the data and identify distinct clusters of genes with comparable expression profiles [26].

### Gene enrichment analysis

Changes in gene expression were visualized using GeneSpring GX version 10.0 (Agilent Technologies, Santa Clara, CA, USA) and this tool was also used to show the identified gene clusters. An enrichment analysis was performed for the gene lists of each cluster using the DAVID functional enrichment tool [27]. Default settings for enrichment analysis in DAVID were used. The total list of genes was used as input and the most enriched functional gene sets (based on Gene Ontology ‘protein domains and pathways’) are reported. These functional gene sets contain at least three genes from a particular gene cluster and are more enriched in the cluster than in the input data set (% genes in cluster ≥ 1.3 x % genes in input gene list).

To define the transcription factors that are responsible for control of a particular cluster of genes, the genes of each cluster were subsequently analyzed in Bibliosphere (Genomatix GmbH, Munich, Germany) with respect to a) shared transcription factor binding sites in their promoter regions and b) co-citation analysis (level B2, co-citation restricted to sentences with a function word). Promoters were defined as 500 bp upstream and 100 bp downstream of the Transcription Start Site of the gene transcript. Default settings of the software were used to perform an overall analysis of the promoters of the genes for common transcription factors. The following criteria were used to define the key transcription factors: a) the transcription factor binding sites have to be present in at least three genes of a cluster and b) are more frequently found in the genes belonging to the cluster of interest than in the total list of input genes (%genes in cluster ≥ 1.3 x % genes in input gene list).

### Comparison of gene expression changes in adipose tissue and liver

Liver and white adipose tissue (WAT) were compared with respect to differentially expressed genes (DEGs relative to the zero time point) and Venn diagrams were prepared to illustrate overlapping genes. A cutoff of FDR P-value < 0.05 was used to define DEGs in both tissues.

The upstream regulator analysis function of Ingenuity Pathway Analysis (IPA) software and the Ingenuity knowledge base were used to analyze the relationship between upstream transcription factors and expression changes of target genes. To test whether a particular transcription factor identified in WAT was also involved in the liver, we analyzed the hepatic transcriptome for differentially expressed target genes of this transcription factor. A P-value P<0.05 indicated that more liver target genes were differentially expressed than expected by chance. Ingenuity Pathway Analysis was also used to test whether a particular transcription factor is activated (positive Z-score >2) or inhibited (negative Z-score <-2) based on the direction of gene expression changes of its target genes.

### Transcription factor analysis

Biochemical transcription factor activity was determined in liver homogenates essentially as previously described [13,28], using TransAM® kit Hnf4α (no. 46296, Active Motif, Europe, Rixensart, Belgium). Briefly, liver homogenates were prepared using the Nuclear Extract Kit (no. 40010, Active Motif, Rixensart, Belgium). Equal amounts of protein (10 μg/well) of the liver homogenates were used to determine the amount of active transcription factor. Control tissues of reference mice on chow were used to correct for the effect of aging.

## Results

### HFD feeding of APOE*3Leiden mice results in obesity and onset of white adipose tissue inflammation

**Figure 1:**
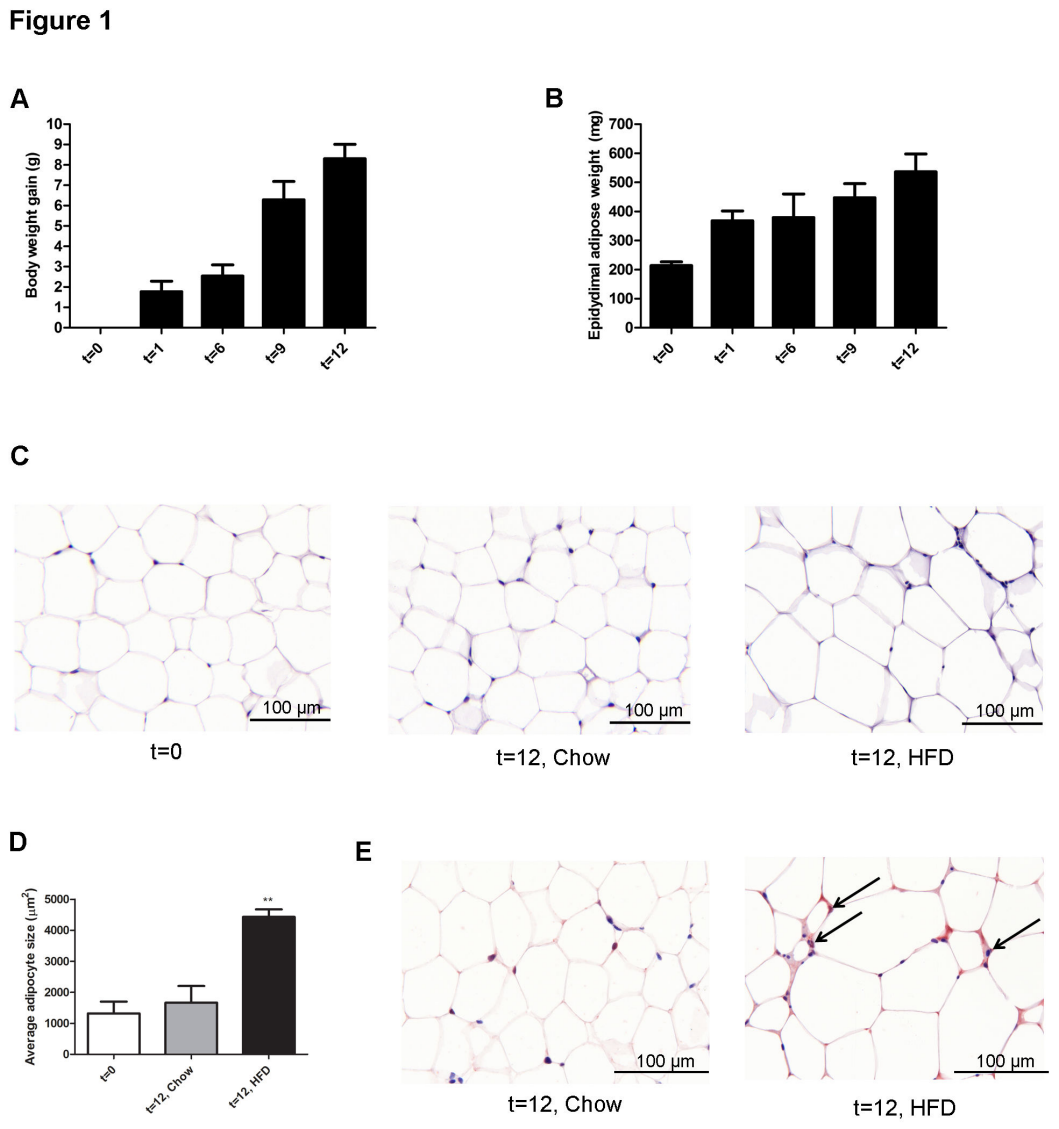
HFD feeding leads to obesity and onset of adipose tissue inflammation. APOE*3Leiden transgenic mice were fed a HFD for 12 weeks and sacrificed at the time points indicated. The average body weight at the start (t=0) of HFD feeding was 29.2 g. **A**, Body weight gain over time. **B**, Mass of the epididymal adipose tissue depot during obesity development. Data are presented as mean ± SEM. **C**, Histological analysis of adipose tissue at start (t=0) and after 12 weeks of HFD or chow feeding (reference for the effect of aging). **D**, HFD feeding results in adipocyte hypertrophy. Computer-assisted quantification of average adipocytes size (P<0.05). **E**, Marked accumulation of CCR2 positive cells (arrows) in the HFD fed group.

APOE*3Leiden mice had an average body weight of 29.2 ± 2.6 g at the start of the experiment (t=0). Animals became obese during HFD feeding and gradually gained 8.30 ± 2.0 g of weight during the experimental period of 12 weeks (Figure 1A) while body weight of control mice on chow remained stable (0.23 ± 0.63 g weight gain; not shown). The daily energy intake per mouse was comparable between the groups fed HFD (15.0 ± 0.9 kcal/day) and chow (14.6 ± 3.0 kcal/day). The HFD-evoked increase in body weight was accompanied by an increase in WAT mass as exemplified by epididymal fat mass (Figure 1B). Histological analysis of epididymal WAT revealed a significant increase in adipocyte size upon HFD feeding relative to chow-fed controls (4431 ± 140 versus 1665 ± 310 µm^2^; P<0.005) demonstrating adipocyte hypertrophy during fat accumulation and obesity development (Figure 1C/1D). In HFD fed mice, immune cells accumulated in WAT at 12 weeks and first crown-like structures were observed (Figure 1C/1E) pointing to an onset of WAT inflammation. Immunochemical analysis demonstrated that accumulating cells in HFD-treated mice were Ccr2-positive (Figure 1E) while Ccr2-positive cells were hardly found in age-matched chow control mice. Together, these data demonstrate that 12 weeks of HFD feeding in ApoE*3Leiden mice resulted in metabolic changes (lipid storage and hypertrophy) as well as onset of WAT inflammation.

### Identification of genes with a similar time profile during HFD feeding

**Figure 2:**
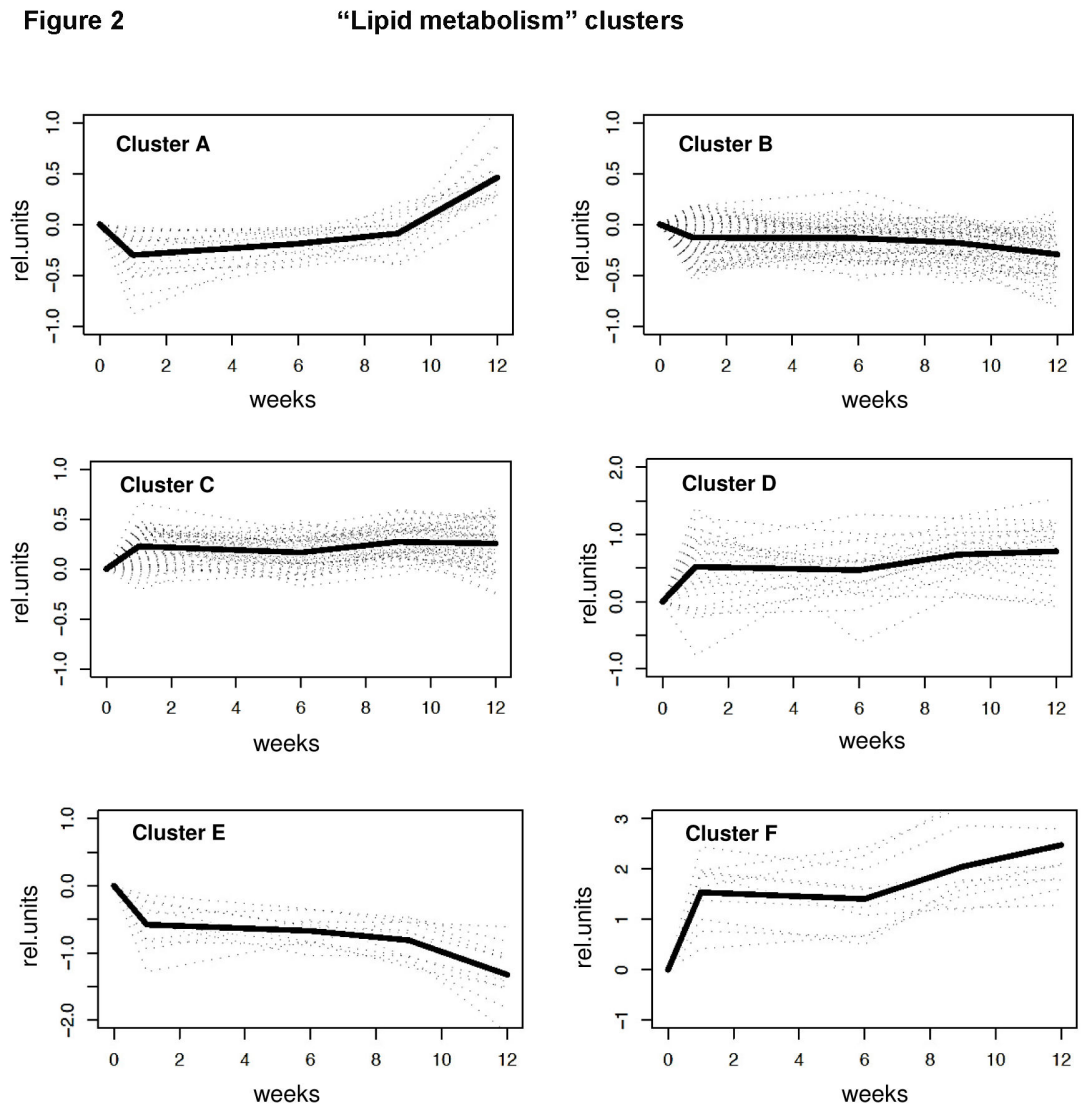
Cluster analysis of genes of lipid metabolism. Bayesian cluster analysis of genes with ‘lipid metabolism’ gene ontology resulted in 6 clusters (A, B, C, D, E, and F) with distinct time profiles. Individual gene expression profiles are shown as dotted lines. The bold line represents the cluster average.

**Figure 3:**
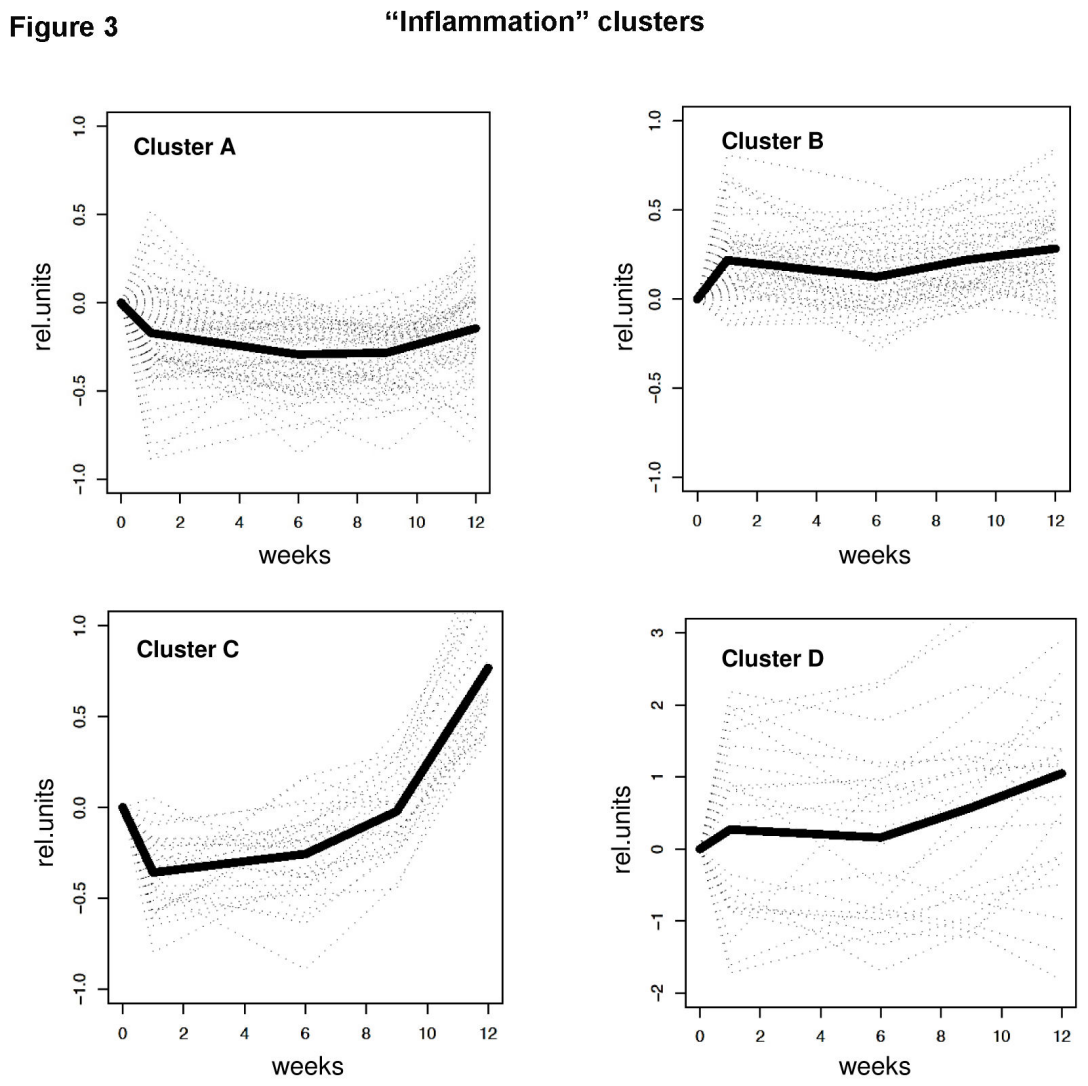
Cluster analysis of inflammatory genes. Bayesian cluster analysis of genes with ‘inflammation’ gene ontology resulted in 4 clusters A, B, C and D with distinct time profiles. Individual gene expression profiles are shown as dotted lines. The bold line represents the cluster average.

To gain insight into the global effects of HFD feeding on metabolism and inflammation in WAT, two sets of genes (i.e. 235 genes with ‘lipid metabolism ontology’ and 216 genes with ‘inflammation ontology’ as defined in Table S2 and Methods) were subjected to Bayesian hierarchical clustering analysis. In this analysis, the individual genes were grouped into gene clusters based on a concerted dynamical expression over time. Genes with a comparable expression pattern across time grouped into specific clusters: Six clusters of lipid metabolism genes (Figure 2) and four clusters of inflammatory genes (Figure 3) were defined. Each cluster showed a distinct and specific time profile suggesting that genes within a cluster share common transcriptional regulation.

Overall, HFD feeding had an early effect on the genes of lipid metabolism and most gene expression changes already occurred within the first week. The majority of the genes of ‘lipid metabolism’ clustered in cluster B and C (165 out of 235). After a slight adjustment in gene expression in week 1, these genes hardly changed over time (Figure 2). The expression changes were somewhat more pronounced in other lipid gene clusters (A, D, E and F), but the main effect also occurred in the first week. It is striking that most ‘lipid metabolism’ genes are rapidly adjusted in the first week and do not adapt to any further extent at the later time points, even when WAT hypertrophy and inflammation are developing. Table 1 shows that the genes of clusters with somewhat more dynamic expression patterns can be assigned to specific biological processes (gene enrichment analysis). For instance, the genes of cluster A were associated with sphingolipid and ceramide metabolism.

**Table 1:** Genes of lipid metabolism with dynamic changes over time.

**Cluster**	**Pattern of time profile**	**Enrichment analysis of biological processes**	**TF**
Cluster A: 20 genes	slight decrease in week 1;	Sphingolipid metabolic process	Jun
	slight increase > week 9	Ceramide metabolic process	Sp1
			Stat1
			Nfĸb1
			Pparγ
Cluster D: 29 genes	slight increase in week 1	Steroid biosynthetic process	Pparα
		Cholesterol biosynthetic process	Nfĸb1
		Cholesterol metabolic process	Pparγ
		Isoprenoid metabolic process	
		Oxidoreductase activity	
Cluster E: 11 genes	Continuous decrease	Steroid metabolic process	Esr1
		Hormone metabolic process	Stat5β
		Reproduction	
		Oxidoreductase activity	
Cluster F: 10 genes	pronounced increase in	Cholesterol absorption	Srebf2
	week 1, then slight increase	Cholesterol metabolic process	Nr1h2
		PPAR signaling pathway	Pparγ
		Lipid binding	Srebf1
		Lipid transport	Hnf4α
		Lipoprotein metabolic process	Sp1
		Glucose metabolic process	Nr1h3
			Nr5a1

Only the genes of cluster A, D, E and F show dynamic changes in expression during the study period. The pattern of the expression changes is described in the second column. Gene clusters are associated with specific biological processes (obtained by gene enrichment analysis) and clustered genes share common transcriptional regulators. These common transcription factors were predicted by promoter analysis and are listed in the last column.

In contrast to the genes involved in lipid metabolism, the mRNA expression level of inflammatory genes increased markedly after week 6 (inflammation clusters C and D in Figure 3). This suggests that factors encoded by these genes may reflect or contribute to the observed onset of WAT inflammation. Indeed, among the upregulated genes were the complement factors C1qa, C1qb, C1qc, C3a receptor-1 and C5a receptor-1, the cytokines Cxcl1/KC, Ccl5/Rantes, Ccl6, Ccl7/Mcp3 and Ccl9/Mrp2, the inflammation markers orosomucoid-1, orosomucoid-3, granzyme A and neutrophil cytosolic factor 1 (Nrf1/p47/phox), the macrophage-associated markers CD11b/Mac1, CD11c, CD18/integrin beta-2, the inflammasome component ASC and the chemokine CXC motive receptor-2 (Ccr2), which is consistent with the observed accumulation of Ccr2-positive cells. Gene enrichment analysis confirmed that these genes belong to processes that promote WAT inflammation such as leukocyte mediated immune response, cytokine activity, complement activation, acute inflammatory response, and cell adhesion (Table 2). Some of the genes encode for inflammatory factors that can be secreted into plasma and may promote inflammation in other tissues.

**Table 2:** Inflammatory genes with dynamic changes over time.

**Cluster**	**Description**	**Enriched processes**	**TF**
Cluster C: 34 genes	slight decrease in first	Inflammatory response;	Pparγ
	week; pronounced	Leukocyte mediated immune	Sfpi1
	increase >week 8	response;	Stat6
		Cytokine activity;	Pax5
		Extracellular region;	Etv6
		B cell mediated immunity;	Pparα
		Complement activation	
Cluster D: 25 genes	slight increases early	Inflammatory response;	Sp1
	in time; pronounced	Acute inflammatory response;	Fos
	increase >week 6	Extracellular space;	Creb1
		Cytokine activity;	Myc
		T cell proliferation;	Vdr
		Cell adhesion	Rarα
			Esr1
			Ar
			Gata1
			Smad2

The genes of cluster C and D are characterized by dynamic changes in expression during HFD feeding. The pattern of the expression changes is described in the second column. Gene clusters are associated with specific biological processes (obtained by gene enrichment analysis) and clustered genes share common transcriptional regulators. Common transcription factors (TF) predicted by promoter analysis are provided in the last column.

### Prediction of transcription factors that control the WAT response to HFD feeding

To identify transcription factors that can orchestrate the observed changes in gene expression profiles in WAT, we analyzed the promoter regions of clustered genes to identify putative common (shared) transcriptional regulators (last column of Tables 1 and 2). Transcriptional binding sites for Jun, Sp1, Stat1, Nfĸb and Pparγ were frequently identified in the promoter regions of the ‘lipid metabolism’ genes in cluster A, i.e. the genes that are related to sphingolipid and ceramide metabolism. Srebf1, Srebf2, Pparγ and Hnf4α were identified as common regulators of the ‘lipid metabolism genes’ of cluster F.

Transcriptional master regulators of the inflammatory genes in cluster D are Pparγ, Sfpi1, Stat6 (cluster C), and Sp1, Fos, Vdr, Esr1, Creb1, Gata1, Smad2 (Table 2). Of note, some transcription factors like Pparγ, Esr1 and Sp1 have a dual role and regulate the expression of genes involved in lipid metabolism and inflammatory genes indicating that these transcription factors operate at the interface of metabolism and inflammation which are thus molecularly interlinked at the level of transcription.

### Key regulators predicted in WAT are also involved in altered liver gene expression

**Figure 4:**
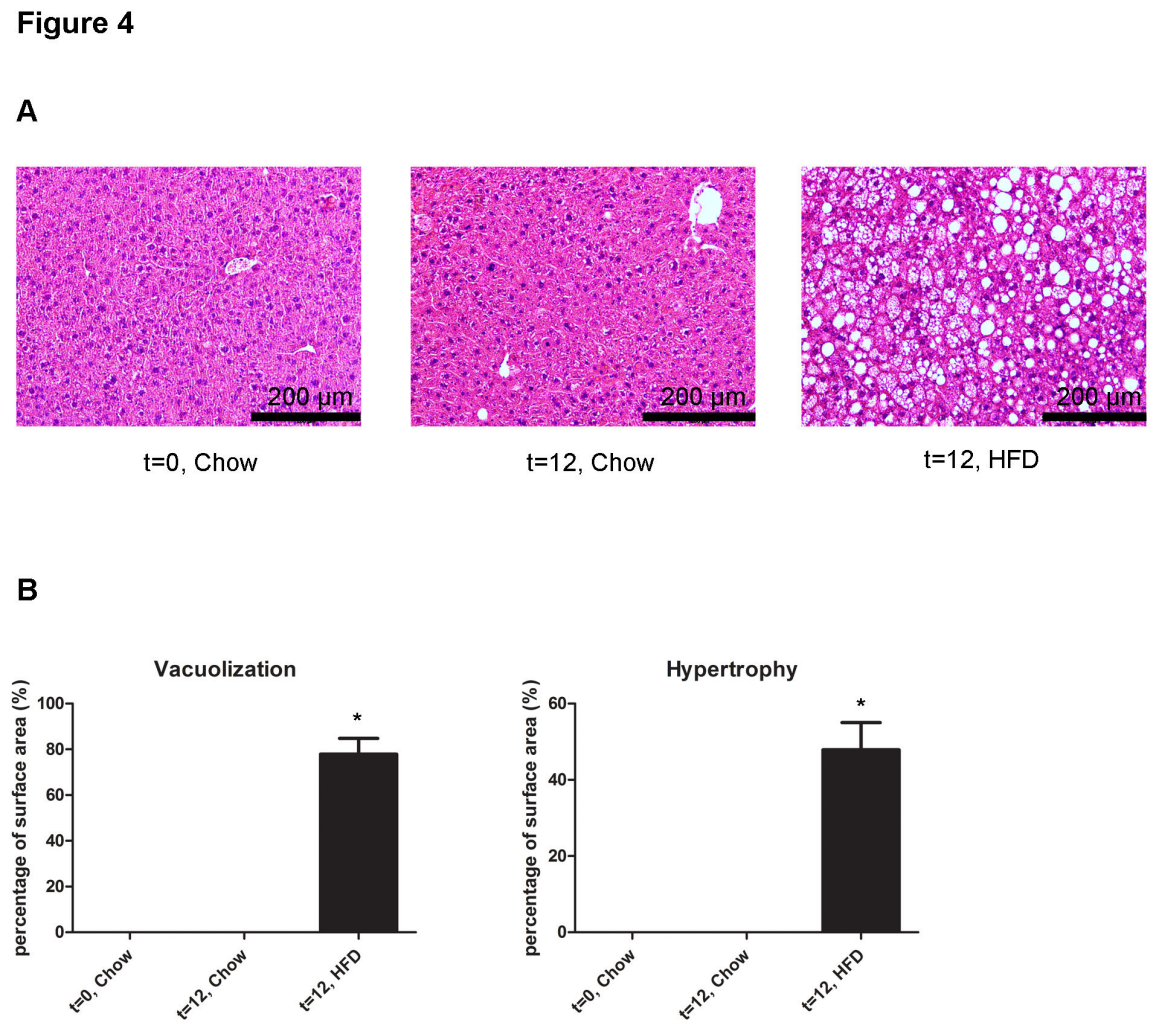
Histological analysis of livers. Hallmarks of non-alcholic fatty liver disease were scored in the livers of the mice that were used for WAT analysis. **A**, Representative photomicrographs of liver cross-sections after 12 weeks of HFD shows pronounced liver steatosis characterized by micro- and macrovacuolization and hepatocellular hypertrophy. **B**, Quantitative analysis of total vacuolization and hypertrophy. Data are presented as mean ± SEM (P<0.05).

**Figure 5:**
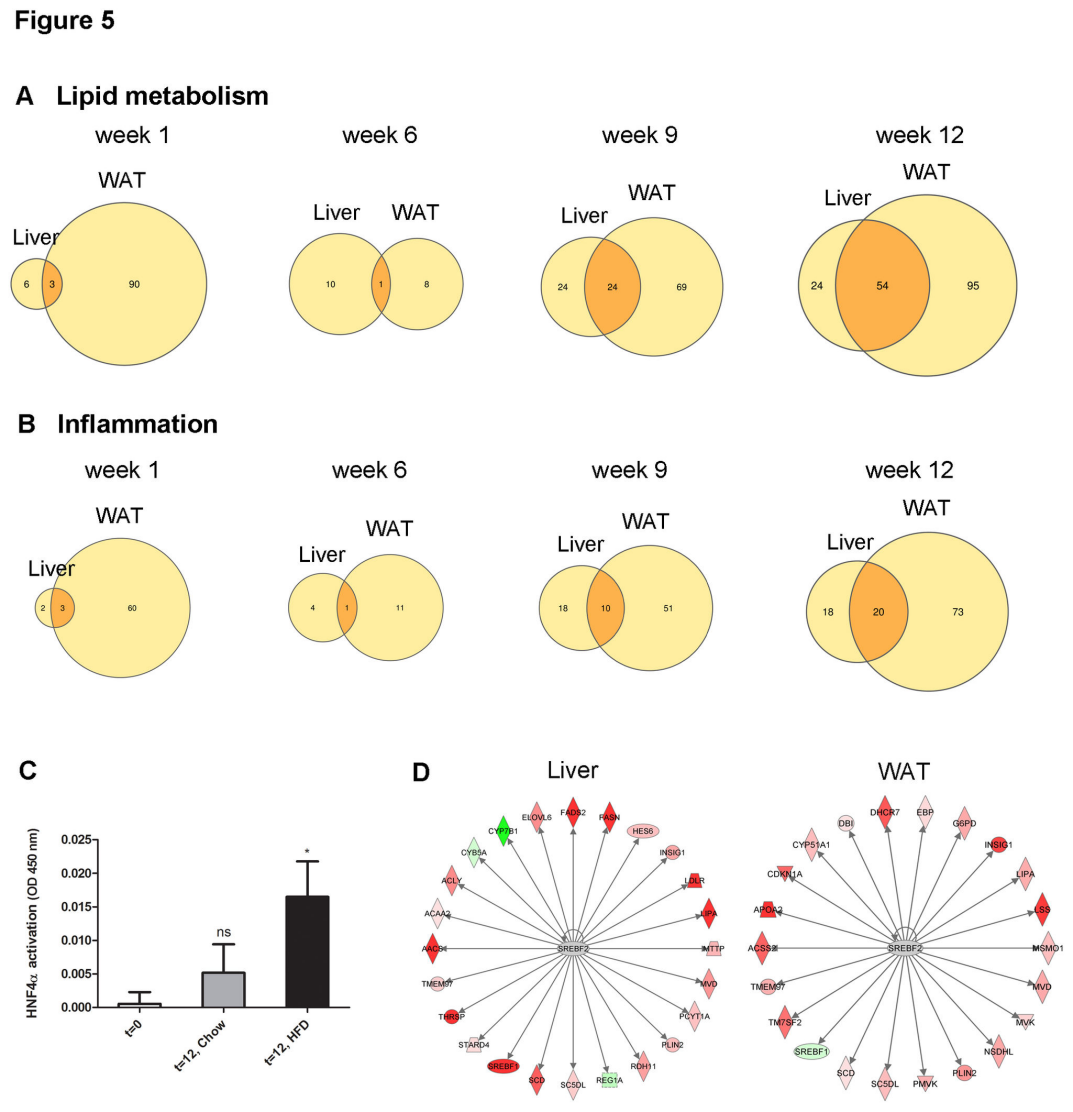
Comparison of gene expression in liver and WAT over time and analysis of transcriptional regulators. Venn diagrams of genes with **A**, ‘lipid metabolism’ gene ontology or **B**, ‘inflammation’ gene ontology. Time course analysis of the genes that were differentially expressed genes at a particular time point. The intersection represents the number of ‘overlapping genes’, ie. genes that were affected in both tissues. **C**, Quantitative analysis of the transcriptional activity of HNF4α by TransAM analysis at t=0 and t=12 weeks of HFD feeding relative to reference mice on chow to correct for aging. *P<0.05. **D**, Differentially expressed target genes of Srebf2 in WAT and liver. Srebf2 is significantly involved in the control of target genes (P<0.05 for both WAT and liver). In both tissues, the calculated Z-score was positive (3.7 for liver and 4.3 for WAT) indicating that Srebf2 is activated. Genes colored in red (green) are upregulated (downregulated).

The livers of the same mice used for the above WAT analysis were examined histologically and using microarrays. Figure 4 shows that HFD feeding but not chow feeding resulted in pronounced micro- and macrovacuolization as well as hepatocellular hypertrophy, demonstrating onset of NAFLD at 12 weeks. Analysis of hepatic gene expression revealed that the genes of lipid metabolism and inflammatory genes were hardly affected until week 6 but thereafter (Figure 5A/5B). To evaluate whether this response to HFD feeding is related to the effects observed in WAT, we compared the gene expression changes in both tissues over time.

Until week 6, only a few differentially expressed genes were found in both tissues, but the number and percentage of common genes (see intersections of Figure 5A and 5B) strongly increased in week 9 and 12, i.e. when WAT becomes overloaded and expression of inflammatory genes of cluster C is observed. Together, these data are in line with the concept that WAT serves as a first buffer to cope with metabolic overload and that the hepatic response is delayed and resembles that of WAT when the storage capacity of WAT is exceeded [5,6]. At week 12, more than 50% of the liver genes with ‘lipid metabolism’ or ‘inflammation’ ontology are also affected in WAT. Analysis of *all* DEGs irrespective of their ontology confirms the relationship between both tissues (245 genes in intersection, 484 liver-specific, 784 WAT-specific in week 12; data not shown).

At the level of transcriptional regulators, the response of both tissues was even more comparable as demonstrated by Bayesian clustering analysis and, as an alternative approach, analysis of target genes. Cluster analysis showed that the gene expression changes until week 9 were modest in liver in comparison with WAT (Figure S1). Similar to WAT, some clusters showed an immediate response to HFD and gene expression did not further change thereafter. Lipid metabolism gene cluster D in the liver had a comparable profile to cluster F of WAT and the predicted transcriptional regulators (Pparγ, Nr1h2, Srebf2, Hnf4α, Nr1h3) were the same. The predicted transcriptional regulators for the inflammatory genes of cluster C in the liver (Ar, Creb1, Esr1, Fos, Myc, Pparγ, Rarα, Sfpi1, Stat6) also overlapped with those predicted for inflammatory genes in WAT. In addition to this, we analyzed the target genes of the master regulators predicted in WAT and tested whether they were differentially expressed in the liver. Statistical testing of the effect on target gene expression showed that the transcription factors Hnf4α, Esr1, Fos, Myc, Pparα, Pparγ, Srebf1 and Srebf2 affected their target genes significantly (all P<0.05), and Creb1 and Jun with borderline significance (P=0.07). Biochemical analysis of the transcriptional binding activity of Hnf4α in liver protein homogenates confirmed its activation (Figure 5C). Of note, a particular transcription factor may affect a different set of genes in WAT and the liver as illustrated in Figure 5D for Srebf2.

Overall, the gene expression effects evoked by HFD in WAT and liver become increasingly comparable showing similar transcriptional responses in both tissues. This indicates that the factors that sense metabolic overload are highly conserved among metabolically active tissues.

## Discussion

The effect of metabolic overload on WAT and liver was analyzed in a mouse model that responds to HFD feeding with WAT expansion, metabolic stress, inflammation and development of NAFLD. Using Bayesian hierarchical clustering we showed that the expression of genes of lipid metabolism is rapidly adjusted upon HFD feeding (already within one week). Cluster analysis revealed that these genes hardly change in expression later in time, despite the observed pronounced WAT expansion and the onset of inflammation from week 6 onward. By contrast, many inflammatory genes strongly increase in their expression at >6 weeks (e.g. inflammatory clusters C and D), and these genes also encode for inflammatory factors that can be secreted into the circulation. This inflammatory response is observed when adipocytes become hypertrophic suggesting that adipose tissue expandability becomes inadequate and the storage capacity of WAT reaches its limit [5,6]. Promoter analysis defined a rather small set of about 25 transcriptional master regulators including Pparγ, Hnf4α, Sp1, Jun/Fos, Esr1, Srebf2, Nr1h2, Sfpi1, Fos, Smad2, Sp1, Gata1 that orchestrate the adaptation of lipid metabolism and induce inflammation. Some of the identified transcription factors (Pparγ, Esr1, Jun/Fos) control the adjustment of lipid metabolism-related and inflammatory genes supporting the view that metabolism and inflammation are molecularly interlinked in WAT [29].

Notably in liver, only a small number of genes of lipid metabolism and inflammation are affected up to week 6. Thereafter, when WAT has become inflamed, a marked increase in the number of differentially expressed genes involved in hepatic lipid metabolism and inflammation was observed. Comparison of liver and WAT revealed a remarkable overlap in gene expression and transcriptional regulation at >6 weeks. Together this shows that HFD feeding results in rapid adaption of WAT lipid metabolism which is not further adjusted during fat storage. When WAT inflammation begins, the gene expression and transcriptional responses of WAT and liver start to resemble each other. This indicates that the fundamental principles of how metabolically active organs cope with HFD overload are conserved.

We found that Pparγ [30], Srebf1 and Srebf2 [31], and Nr1h3/Nr1h2 (also referred to as Lxrα/Lxrβ) [32,33] may explain the observed gene expression changes. Indeed, these transcription factors are well-established regulators of lipid metabolism and their identification confirms the validity of the approach applied. The ‘lipid metabolism’ genes with the largest changes grouped in lipid gene cluster F. Genes in this cluster are not only involved in lipid metabolism but also in lipid absorption and glucose metabolism and could reflect a reprogramming of WAT from early time points onward to cope with HFD overload. Potential transcription factors regulating this reprogramming include Srebf1, Srebf2, Pparγ, Lxrα, Lxrβ and Hnf4α. In a previous study, we indeed identified transcription factor HNF4α as a regulator of energy metabolism in human adipose tissue [34], and activation of LXRα and LXRβ has been shown to affect lipid and glucose metabolism as well as the inflammatory state simultaneously [28,35].

We also identified several transcription factors typically associated with inflammation (Jun, Stat1, Stat5β) in the promoter elements of genes associated with general sphingolipid metabolism and, more specifically, ceramide metabolism. This finding supports the notion that molecular links exist between lipid metabolism and inflammatory signaling cascades and that these processes are interlinked and hence, may influence each other [23,29,36]. Boini and coworkers have shown that HFD-treated mice have increased levels of ceramide in WAT and in plasma [37,38]. In another study, the ceramide concentrations in human WAT were positively correlated with the inflammatory state of the tissue, independent of obesity [39].

The inflammatory/immune response genes of the inflammatory gene clusters C and D showed a pronounced increase in expression from week 6 onward. One of the genes encodes for Ccl5/Rantes which promotes macrophage recruitment in adipose tissue [40]. Indeed, the gene expression levels of CD11b/Mac1, a marker expressed on macrophages and neutrophils, were also increased showing a similar time pattern. Of note, the expression of another inflammatory gene, Cxcl1/KC, intensified also from week 6 onwards. This coincides with the development of insulin resistance in WAT of APOE*3Leiden mice under the experimental conditions employed herein [13]. Cxcl1/KC stimulates the infiltration of neutrophils into WAT [41] and represents the mouse ortholog of human interleukin-8, but the exact role of this factor in the pathogenesis of insulin resistance remains to be established. The observation that neutrophil cytosolic factor 1 (Nrf1/p47/phox) expression levels also increase suggests that (infiltrating) neutrophils may have a role early in the disease process. Of note, also the expression levels of granzyme A, a protease present in granules of cytotoxic T-cells and NK cells, increased strongly from week 6 onwards. Because immune cells accumulate in WAT during HFD feeding, it is thus likely that changes in inflammatory gene expression may, at least partly, be a reflection of the changes in cellular composition of the tissue. Interestingly, we also found a gradual increase in expression of the inflammasome adaptor ASC. ASC is necessary for assembly of inflammasome complexes, which activates the inflammatory cytokines IL1 and IL18 from their propeptides in response to saturated fatty acid overload, thereby linking lipid metabolism and inflammation and promoting the development of insulin resistance in T2DM [42]. Promoter analyses of clustered inflammatory genes revealed that a large number of these genes share Fos, Smad2, Stat6 and Pparα as transcriptional regulators, which is in accordance with their established roles in inflammatory signaling cascades [43-46].

In several clusters, the transcriptional regulators Pparγ, Sp1, estrogen receptor 1 (Esr1) and Jun/Fos were identified as central underlying transcriptional regulators that may explain the gene expression changes of both lipid metabolism genes and inflammation-related genes. In accordance with their suggested overarching role, Pparγ, Sp1 and Esr1 are indeed involved in cell differentiation, cell cycle and growth and immune response processes [23,30,47,48]: Pparγ is implicated in adipogenesis and insulin signaling of adipocytes as well as in the control of the inflammatory state of infiltrating monocytes/macrophages [49]. Esr1 forms complexes with DNA-bound Sp1 to regulate the transcription of low density lipoprotein receptor (LDLR) [50], retinoic acid receptor-alpha (Rarα) [51] and c-Fos [52]. Another transcription factor that may constitute a link between lipid metabolism and inflammation is c-Jun as established previously for WAT and liver [23,36,53].

A limitation of the present study is that the transcriptional regulator prediction method predicts the binding of the transcription factors only from the existence of the binding motifs in the regulatory elements of genes, i.e. it employs available knowledge about the regulation of these genes. Although the results of this bioinformatical approach are certainly indicative, the involvement of many of these transcription factors has not been experimentally proven under the experimental conditions employed and there is a recent recognition that only a small portion of the putative motif may actually be occupied by the transcription factors based on recent ChIP-seq studies.

Because WAT and liver tissue have evolved from common ancestral structures (mesoderm) it has been proposed that they may share similar functional units to control key metabolic and immune processes [29,54]. Indeed, our results show that the factors important for regulation of gene expression in WAT were also affected in liver, suggesting a considerable consistency between both responses to HFD-induced metabolic overload. Some of the master regulators identified in WAT in the present study (Jun, Fos, Rarα, Pparα, Stat1, Stat5, Sp1) were also reported to control liver lipid metabolism and/or the inflammatory responses of the liver in experimental diet-induced cardiovascular disease [23]. A tight relationship between WAT dysfunction and the pathogenesis of NAFLD has been reported recently [55], suggesting comparable control of inflammatory gene expression in metabolically active organs [29]. This interrelationship could possibly be exploited in the future to monitor the condition of the liver via biopsies taken from WAT because they are more accessible. Our findings correspond with the view that the control mechanisms of metabolic and inflammatory homeostasis in WAT and liver share similarities and that a distortion of the mechanisms that control metabolic adaptation may also affect the inflammatory tone of a tissue [9,29].

Collectively, this study demonstrates that high fat feeding evokes an immediate, stable response of lipid metabolism genes. Later in time, when the storage capacity of WAT becomes limited, inflammatory genes are induced in WAT (>6 weeks). When WAT began, genes of lipid metabolism and inflammation also became affected in corresponding livers. The hepatic response to HFD, in particular the underlying transcriptional responses, were remarkably similar to those detected in WAT. In all, WAT and liver respond to metabolic overload by adaptations in expression of (clusters of) genes controlling lipid metabolism and inflammatory processes in an orchestrated and interrelated manner.

## Supporting Information

Figure S1 **Lipid metabolism and inflammation gene clusters in liver.** Lipid metabolism gene clusters (A,B,C,D). A, No dynamics. B, No dynamics. C, Dynamics but no common transcriptional regulators. D, Dynamic changes similar to cluster F of WAT. Common predicted regulators: Pparγ, Nr1h2, Srebf2, Hnf4α, Nr1h3. Inflammation clusters (E,F,G). E, No dynamics. F, Hardly dynamics, no common transcriptional regulators. G, Dynamic changes of individual genes; predicted transcriptional regulators Stat6, Rarα, Ar, Esr1, Fos, Sfpi1, Pparγ, Myc, Creb1 have overlap with those identified in WAT.
(PPTX)

Table S1 **Diet composition.**
(DOCX)

Table S2 **Lipid metabolism and inflammation gene sets in WAT.** The authors thank Nicholas Heard for help with the clustering algorithm. We thank Erik H. Offerman, Wim van Duyvenvoorde and Karin Toet for technical assistance.
(XLS)
