# Supplementary material for: Coordinated and Interactive Expression of Genes of Lipid Metabolism and Inflammation in Adipose Tissue and Liver during Metabolic Overload
Source: PLoS One. 2013 Sep 25;8(9):e75290. doi: 10.1371/journal.pone.0075290 (PMC3783477; doi:10.1371/journal.pone.0075290)
Supplement: Figure S1 — Lipid metabolism and inflammation gene clusters in liver. Lipid metabolism gene clusters (A,B,C,D). A, No dynamics. B, No dynamics. C, Dynamics but no common transcriptional regulators. D, Dynamic changes similar to cluster F of WAT. Common predicted regulators: Pparγ, Nr1h2, Srebf2, Hnf4α, Nr1h3. Inflammation clusters (E,F,G). E, No dynamics. F, Hardly dynamics, no common transcriptional regulators. G, Dynamic changes of individual genes; predicted transcriptional regulators Stat6, Rarα, Ar, Esr1, Fos, Sfpi1, Pparγ, Myc, Creb1 have overlap with those identified in WAT. (PPTX) [file pone.0075290.s001.pptx]

## Slide 1
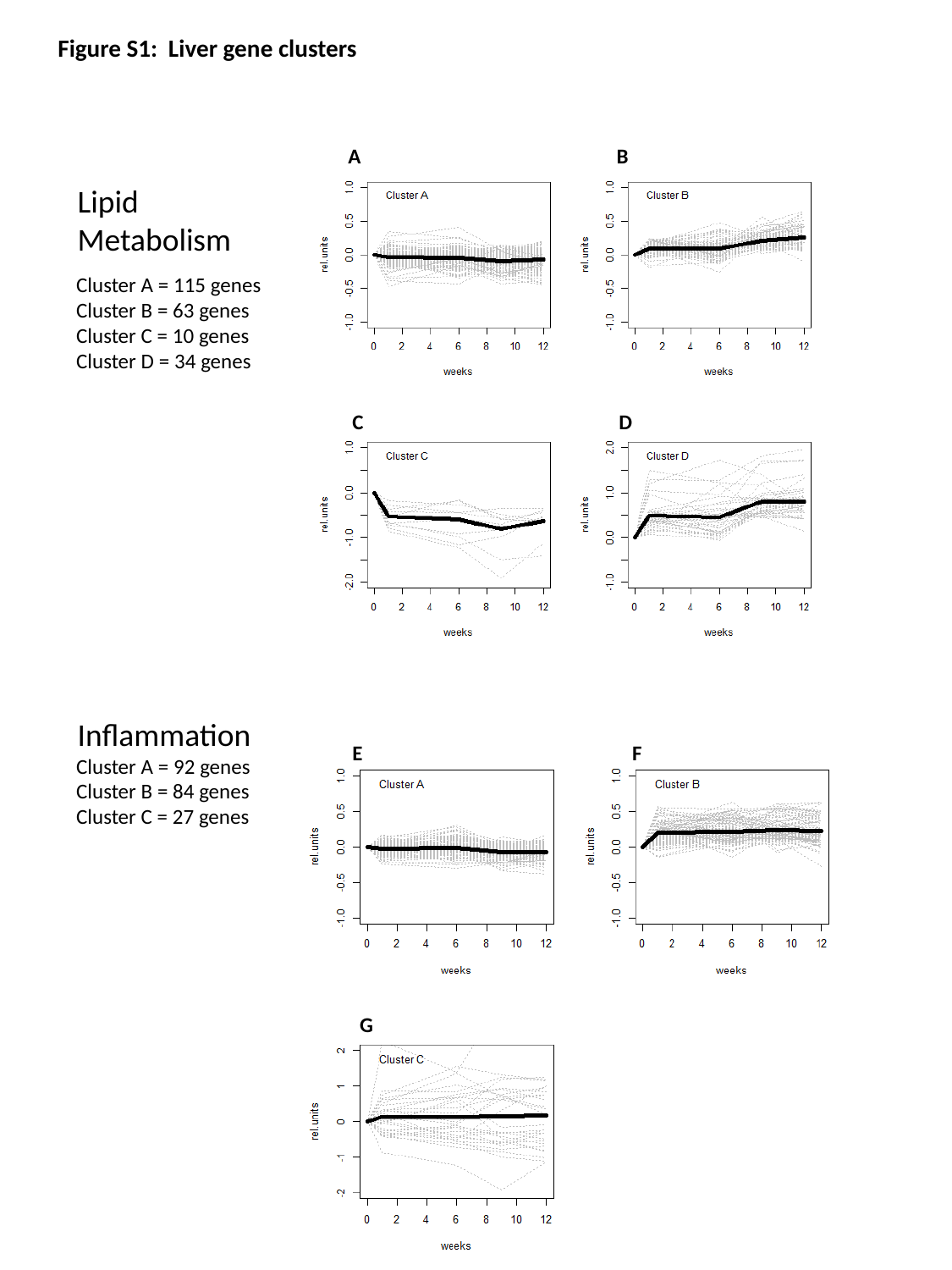

Figure S1: Liver gene clusters
A
B
Lipid
Metabolism
Inflammation
Cluster A = 115 genes
Cluster B = 63 genes
Cluster C = 10 genes
Cluster D = 34 genes
Cluster A = 92 genes
Cluster B = 84 genes
Cluster C = 27 genes
D
C
E
F
G
