# Supplementary figures and images for: Coordinated and Interactive Expression of Genes of Lipid Metabolism and Inflammation in Adipose Tissue and Liver during Metabolic Overload
Source: PLoS One. 2013 Sep 25;8(9):e75290. doi: 10.1371/journal.pone.0075290 (PMC3783477; doi:10.1371/journal.pone.0075290)

**
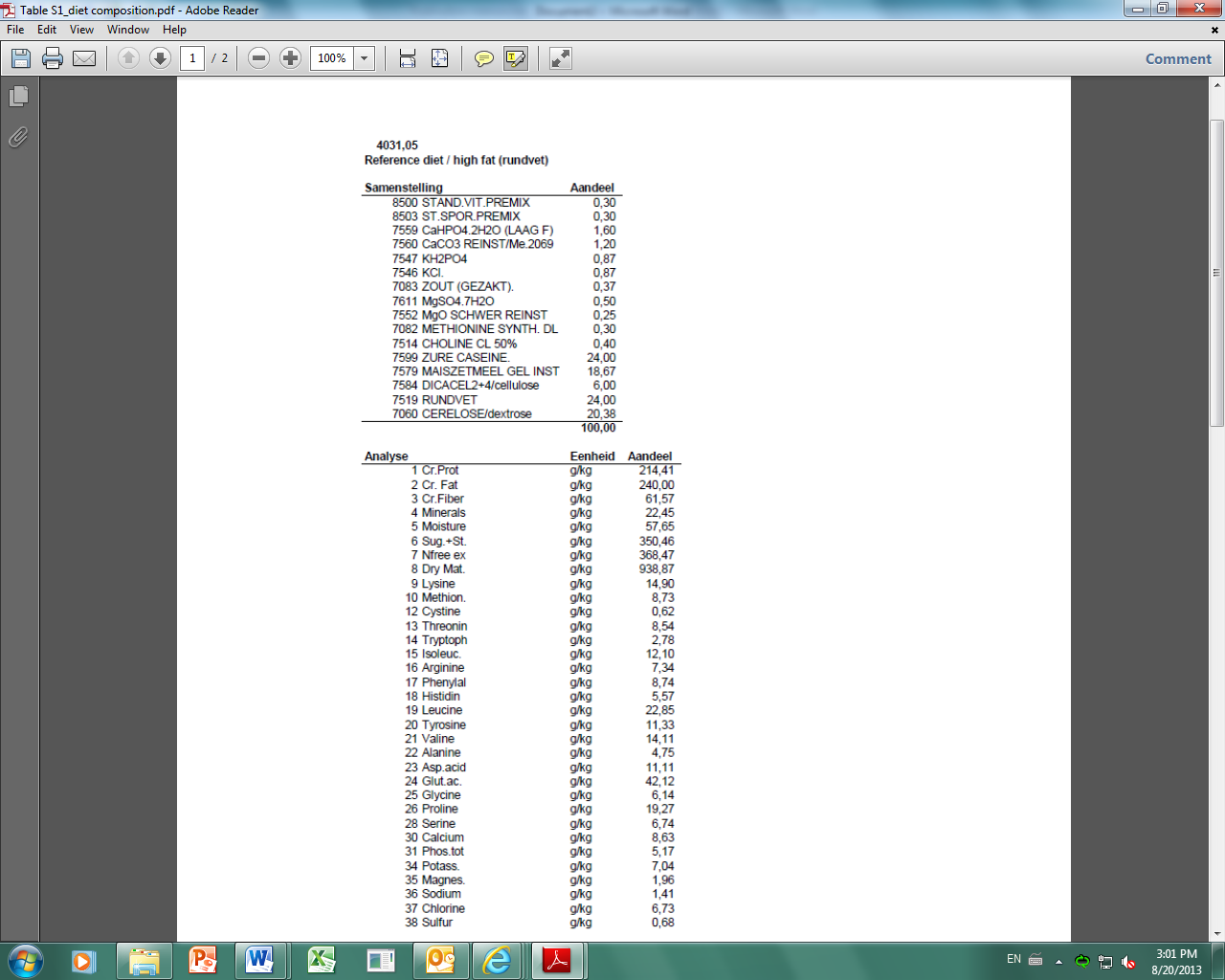
Table S1 Diet composition**


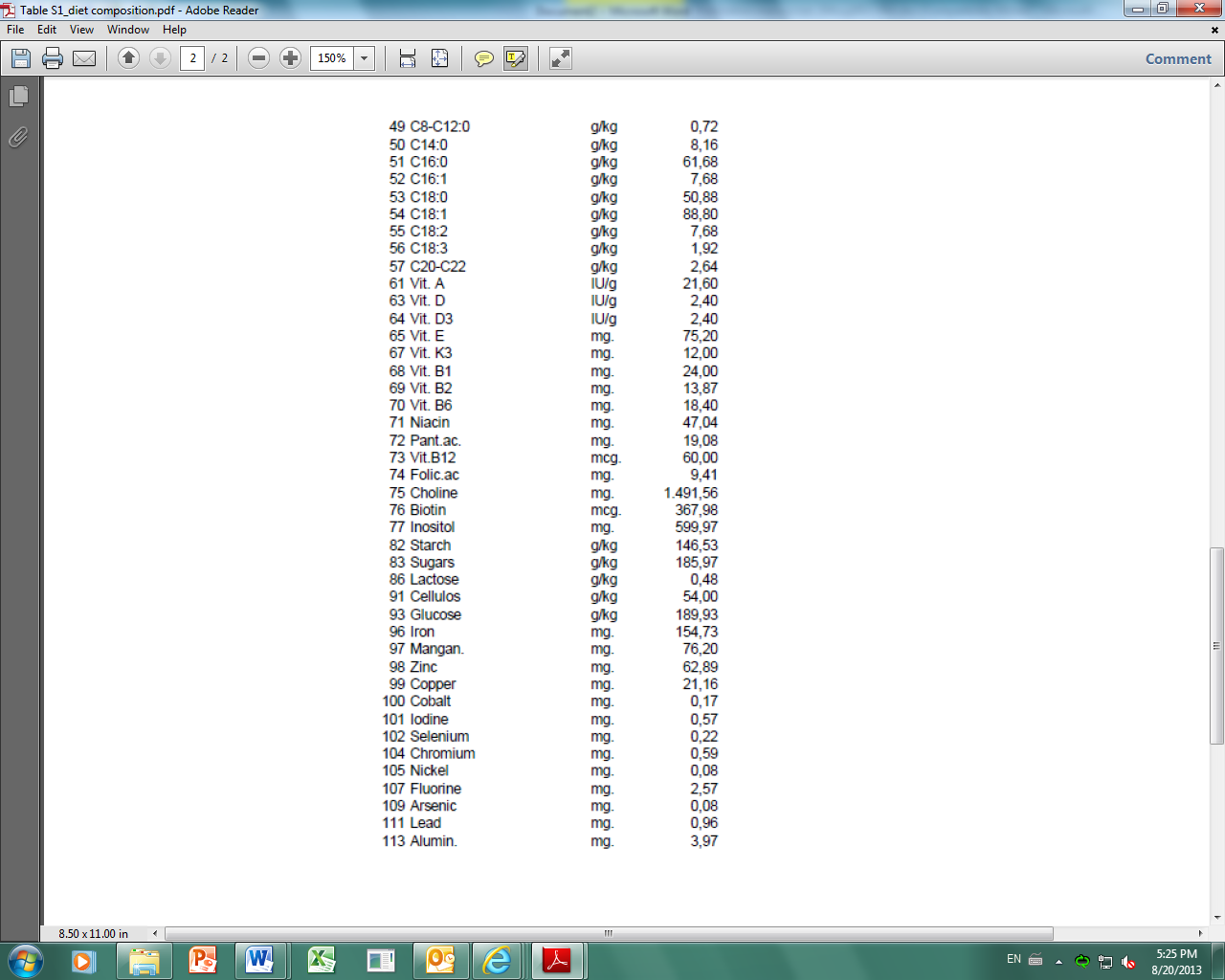

Supplement: Table S1 — Diet composition. (DOCX) [file pone.0075290.s002.docx]
